# Monitoring of transcriptional regulation in *Pichia pastoris *under protein production conditions

**DOI:** 10.1186/1471-2164-8-179

**Published:** 2007-06-19

**Authors:** Brigitte Gasser, Michael Maurer, Jari Rautio, Michael Sauer, Anamitra Bhattacharyya, Markku Saloheimo, Merja Penttilä, Diethard Mattanovich

**Affiliations:** 1 University of Natural Resources and Applied Life Sciences Vienna, Department of Biotechnology, Institute of Applied Microbiology, Muthgasse 18, A-1190 Vienna, Austria; 2 School of Bioengineering, University of Applied Sciences FH Campus Wien, Muthgasse 18, A-1190 Vienna, Austria; 3 VTT Technical Research Centre of Finland, P.O. Box 1000, 02044 VTT, Espoo, Finland; 4 Integrated Genomics, Inc., 2201 W. Campbell Park Drive, Suite 15, 606012-3547 Chicago, IL, USA

## Abstract

**Background:**

It has become evident that host cells react to recombinant protein production with a variety of metabolic and intrinsic stresses such as the unfolded protein response (UPR) pathway. Additionally, environmental conditions such as growth temperature may have a strong impact on cell physiology and specific productivity. However, there is little information about the molecular reactions of the host cells on a genomic level, especially in context to recombinant protein secretion. For the first time, we monitored transcriptional regulation of a subset of marker genes in the common production host *Pichia pastoris *to gain insights into the general physiological status of the cells under protein production conditions, with the main focus on secretion stress related genes.

**Results:**

Overexpression of the UPR activating transcription factor Hac1p was employed to identify UPR target genes in *P. pastoris *and the responses were compared to those known for *Saccharomyces cerevisiae*. Most of the folding/secretion related genes showed similar regulation patterns in both yeasts, whereas genes associated with the general stress response were differentially regulated. Secretion of an antibody Fab fragment led to induction of UPR target genes in *P. pastoris*, however not to the same magnitude as Hac1p overproduction. Overexpression of *S. cerevisiae *protein disulfide isomerase (*PDI1*) enhances Fab secretion rates 1.9 fold, but did not relief UPR stress. Reduction of cultivation temperature from 25°C to 20°C led to a 1.4-fold increase of specific product secretion rate in chemostat cultivations, although the transcriptional levels of the product genes (Fab light and heavy chain) were significantly reduced at the lower temperature. A subset of folding related genes appeared to be down-regulated at the reduced temperature, whereas transcription of components of the ER associated degradation and the secretory transport was enhanced.

**Conclusion:**

Monitoring of genomic regulation of marker genes with the transcriptional profiling method TRAC in *P. pastoris *revealed similarities and discrepancies of the responses compared to *S. cerevisiae*. Thus our results emphasize the importance to analyse the individual hosts under real production conditions instead of drawing conclusions from model organisms. Cultivation temperature has a significant influence on specific productivity that cannot be related just to thermodynamic effects, but strongly impacts the regulation of specific genes.

## Background

Heterologous protein production processes in the methylotrophic yeast *Pichia pastoris *became increasingly important in the last decade. Although *P. pastoris *is known as a highly efficient expression system, there is only little knowledge about the physiology and the genetics lying underneath.

Especially the production of complex proteins has turned out to have a rather low success rate (see reviews by [1,2]). Several physiological studies have demonstrated that many processes, including stress responses to environmental factors, and protein folding/aggregation and secretion are highly interrelated. Among the environmental factors influencing protein expression and secretion, pH, osmolarity, oxygen availability and temperature appear to be particularly important (recently reviewed by [3]).

During the development of *P. pastoris *strains secreting either the human serine protease trypsinogen [4], *Rhizopus oryzae *lipase [5,6], or the Fab fragments of the human monoclonal anti-HIV1 antibody 2F5 [7] and its anti-ideoptype 3H6 [8], limitations in folding and/or secretion became obvious. Using immunofluorescent staining and flow cytometry techniques we could detect intracellularly retained product, which was found to be located within the membrane fraction of cell lysates (containing compartments of the secretory pathway such as the ER and the Golgi), and a concomitant increase in the levels of intracellular BiP (binding protein), which is described as a signal molecule for unfolded protein response (UPR) (for reviews see [9-11]). On the other hand it has been shown that constitutive induction of the UPR pathway by overexpression of the transcriptional activator Hac1p can promote folding and secretion of heterologous proteins in several hosts, including *Saccharomyces cerevisiae *[12], *Aspergillus niger *var. *awamori *[13] and *P. pastoris *[7].

To our knowledge there exists no report about the regulation of gene expression upon activation of the UPR pathway in *P. pastoris *so far. We attempted to analyse these responses in more detail and to determine the possible induction of the UPR as result of the overexpression of heterologous proteins on a transcriptional level. The rapid transcriptional profiling method TRAC (transcript analysis with aid of affinity capture) that has been successfully implemented for *T. reesei *[14,15] was employed in this study to monitor the levels of a subset of mRNAs coding for UPR-regulated and stress-connected genes in *P. pastoris*.

While a lot of data has been collected regarding the regulatory events as a reaction to temperature changes, there is not much information on the true cellular reaction particularly in context of heterologous protein expression. The optimum growth temperature for *P. pastoris *is usually defined as 28–30°C, however, it is well established that a reduction of the temperature to e.g. 25°C can significantly improve recombinant protein productivity without hampering growth [16]. Jahic *et al. *[17] suggested that lower temperature leads to a reduced death rate, and hence a lower amount of host cell proteases in the supernatant. While this seems plausible, it should be noted that during our work we did not find such an influence of temperature on cell death at common growth temperatures [4].

Therefore a deeper understanding of the physiological and molecular links between protein folding and temperature (-adaption/-stress) appears useful.

We investigated production characteristics at 20°C compared to 25°C in chemostat cultivations expressing 2F5 Fab fragment. First results indicated that specific product formation rate was 1.4-fold higher at the lower temperature compared to steady state conditions at 25°C. Therefore transcriptional profiling with the TRAC method was employed to study whether this increase in product secretion is a result of increased levels of UPR-regulated genes or if there are alternative mechanisms lying underneath.

## Results and discussion

### Expression of more than 50 genes was monitored by the TRAC method in four recombinant *P. pastoris *strains and the wild type grown in various conditions

TRAC analysis was performed in duplicates for each sample in two of the authors' laboratories in Finland (VTT) and Austria (IAM). Variation between these duplicate assays (*S*_*MV*_) was 10% on the average, and independent of signal strength as it was reported for *T. reesei *samples by [14].

For the temperature experiments, the ratio of the fluorescence intensity per ng polyA RNA (FL) in 20°C steady state to the FL in 25°C steady state samples was determined individually for each of the two biological replicas. The mean ratio was calculated for each probe, with the standard deviation (*S*_*BV*_) of the ratios being 21% on average. So the overall standard deviation of the temperature experiment (*S*_*A*_) adds up to 25%. The probability (*p*-values) that the FL 20°C does actually differ significantly from FL 25°C was assessed with a Student t-test.

The *P. pastoris *gene annotations in the ERGO genome database (Integrated Genomics; Chicago, IL, [18]) are derived using a number of criteria [19] including orthology to *S. cerevisiae *gene function. Consequently, all the discussion will be based on the respective descriptions (Table 1) given in the Saccharomyces Genome Database [20].

**Table 1:** Functions of the markers used in TRAC analysis.

	**Folding**
CPR5	Cyclophilin type peptidyl-prolyl cis-trans isomerase (ER resident)
ERO1	Pdi oxidase, protein-thiol disulfide exchange; required for oxidative protein folding in the ER
KAR2	Binding protein BiP, ATPase involved in protein import into the ER, acts as a chaperone to mediate protein folding in the ER; regulates the unfolded protein response
PDI1	Protein disulfide isomerase, multifunctional protein resident in the ER lumen, essential for the formation of disulfide bonds in secretory and cell-surface proteins
	**Glycosylation/ER quality control**
CNE1	Calnexin; integral membrane ER chaperone involved in folding and quality control of glycoproteins
DFM1	ER localized derlin-like family member involved in ER stress and homeostasis; not involved in ERAD or substrate retrotranslocation
PMT6	Protein O-mannosyltransferase, transfers mannose from dolichyl phosphate-D-mannose to protein serine/threonine residues of secretory proteins
ROT2	Mannosyl-oligosaccharide glucosidase II, required for normal cell wall synthesis
SEC53	Phosphomannomutase, involved in synthesis of GDP-mannose and dolichol-phosphate-mannose; required for folding and glycosylation of secretory proteins in the ER lumen
	**Secretion**
BMH2	14-3-3 protein, minor isoform; binds proteins and DNA, involved in regulation of many processes including exocytosis and vesicle transport
SAR1	GTPase, GTP-binding protein of the ARF family, component of COPII coat of vesicles; required for vesicle formation during ER to Golgi protein transport
SEC18	ATPase required for vacuole fusion and for ER to Golgi vesicle mediated transport
SEC31	Essential phosphoprotein component (p150) of the COPII coat of secretory pathway vesicles, in complex with Sec13p; required for ER-derived transport vesicle formation
SEC61	Essential subunit of Sec61 complex, forms a channel for SRP-dependent protein import and retrograde transport of misfolded proteins out of the ER
SNC1	Vesicle membrane receptor protein (v-SNARE) involved in the fusion between Golgi-derived secretory vesicles with the plasma membrane
SSO2	Plasma membrane t-SNARE involved in fusion of secretory vesicles at the plasma membrane
	**ERAD (ER-associated protein degradation)**
HRD1	Ubiquitin-protein ligase (EC 6.3.2.19) required for ERAD of misfolded proteins
UBC1	Ubiquitin-conjugating enzyme E2 (EC 6.3.2.19) that mediates selective degradation of short-lived and abnormal proteins; plays a role in vesicle biogenesis and ERAD
	**DNA repair**
RAD16	Subunit of Nucleotide Excision Repair Factor 4
RAD2	Single-stranded DNA endonuclease, cleaves ssDNA during nucleotide excision repair to excise damaged DNA; subunit of Nucleotide Excision Repair Factor 3 (NEF3)
RAD54	DNA-dependent ATPase, stimulates strand exchange by modifying the topology of double-stranded DNA; involved in the recombinational repair of ds breaks in DNA
	**Travers UPR**
ARL3	GTPase of the Ras superfamily, required to recruit Arl1p to the Golgi
RIB1	GTP cyclohydrolase 2
VPS17	Subunit of the membrane-associated retromer complex essential for endosome-to-Golgi retrograde protein transport
	**Core metabolism**
ACS1	Acetyl-coA synthetase isoform which catalyzes the formation of acetyl-CoA from acetate and CoA
CIT1	Citrate synthase, catalyzes the condensation of acetyl coenzyme A and oxaloacetate to form citrate; the rate-limiting enzyme of the TCA cycle
PFK1	Phosphofructokinase alpha subunit
TAL1	Transaldolase, enzyme in the non-oxidative pentose phosphate pathway; converts sedoheptulose 7-phosphate and glyceraldehyde 3-phosphate to erythrose 4-phosphate and fructose 6-phosphate
TKL1	Transketolase, catalyzes conversion of xylulose-5-phosphate and ribose-5-phosphate to sedoheptulose-7-phosphate and glyceraldehyde-3-phosphate in the pentose phosphate pathway; needed for synthesis of aromatic amino acids
	**General stress response**
BFR2	Essential protein possibly involved in secretion; multicopy suppressor of sensitivity to Brefeldin A, homolog of LTV1 (low temperature viability protein)
CUP5	Vacuolar ATP synthase proteolipid subunit (EC 3.6.3.14) required for vacuolar acidification and important for copper and iron metal ion homeostasis
GCN4	Transcriptional activator of amino acid biosynthetic genes in response to amino acid starvation
HEM12	Uroporphyrinogen decarboxylase, catalyzes the fifth step in the heme biosynthetic pathway
HOG1	Mitogen-activated and osmosensing protein kinase, involved in osmoregulation
IMH1	Protein involved in vesicular transport, mediates transport between an endosomal compartment and the Golgi, contains a GRIP domain that interacts with activated Arl1p-GTP to localize Imh1p to the Golgi
KIN2	Serine/threonine protein kinase involved in regulation of exocytosis; localizes to the cytoplasmic face of the plasma membrane
MSN2	Transcriptional activator; activated in stress conditions; binds DNA at stress response elements; response to aging, freezing, osmotic, oxidative stress and glucose starvation
SNZ2/3	Member of a stationary phase-induced gene family, involved in pyridoxine and thiamin biosynthesis
	**Thioredoxin**
TRR1	Cytoplasmic thioredoxin reductase, key regulatory enzyme that determines the redox state of the thioredoxin system, which acts as a disulfide reductase system and protects cells against both oxidative and reductive stress
TSA1	Thioredoxin peroxidase, reduces reactive oxygen, nitrogen and sulfur species using thioredoxin as hydrogen donor
	**Amino acid synthesis**
ARG1	Arginosuccinate synthetase, catalyzes the formation of L-argininosuccinate from citrulline and L-aspartate in the arginine biosynthesis pathway
ARO4	3-deoxy-D-arabino-heptulosonate-7-phosphate (DAHP) synthase, catalyzes the first step in aromatic amino acid biosynthesis
GLT1	NAD(+)-dependent glutamate synthase, synthesizes glutamate from glutamine and alpha-ketoglutarate
	**Glutathione**
GLR1	Glutathione oxidoreductase, converts oxidized glutathione to reduced glutathione
GSH2	Glutathione synthetase, catalyzes the ATP-dependent synthesis of glutathione (GSH) from gamma-glutamylcysteine and glycine; induced by oxidative stress and heat shock
	**Cytosolic hsps**
HSP12	Plasma membrane localized protein that protects membranes from desiccation; induced by heat shock, oxidative stress, osmostress, stationary phase entry, glucose depletion, regulated by the HOG and Ras-Pka pathways
HSP90/82	Cytoplasmic chaperone (Hsp90 family), required for the activation of many key cellular regulatory and signaling proteins, like kinases and transcription factors
SSA4	Cytoplasmic member of the HSP70 family; highly induced upon stress; plays a role in SRP-dependent cotranslational protein-membrane targeting and translocation
SSE1/2	ATPase that is a component of the Hsp90 chaperone complex; binds unfolded proteins; member of the HSP70 family; localized to the cytoplasm
	**Ribosomes**
RPL3	Protein component of the large (60S) ribosomal subunit
RPS23B	Ribosomal protein 28 (rp28) of the small (40S) ribosomal subunit
	**Heterologous protein**
LC	Fab light chain
HC	Fab heavy chain

Functions of the *S. cerevisiae *homologs of the *P. pastoris *gene markers (adapted from *Saccharomyces *Genome Database [20]) assigned in functional categories.

### Effect of Hac1-overexpression on the regulation of marker genes in *P. pastoris*

As the active form of *P. pastoris *Hac1p is not identified yet, the *S. cerevisiae *homolog was used. Cross species functionality of Hac1p has been demonstrated between *S. cerevisiae *and *P. pastoris *[7], and *T. reesei *respectively [12]. Therefore, TRAC analysis of shake flask cultivations of *P. pastoris *strains co-overexpressing *S. cerevisiae *UPR-transcription factor Hac1p was applied to identify UPR-targets in *P. pastoris *and the responses were compared to those observed in *S. cerevisiae *[21]. In that study, *S. cerevisiae *UPR target genes were induced by dithiothreitol (DTT) and tunicamycin, two agents known to interfere with protein folding in the ER.

Both *P. pastoris *GS115, and the protease deficient strain SMD1168, containing *S. cerevisiae HAC1* were analysed, and no difference could be seen in the regulation of the marker genes included in our analysis. Therefore a mean value of these two experiments was compared to untransformed X33 as control strain.

**Figure 1:**
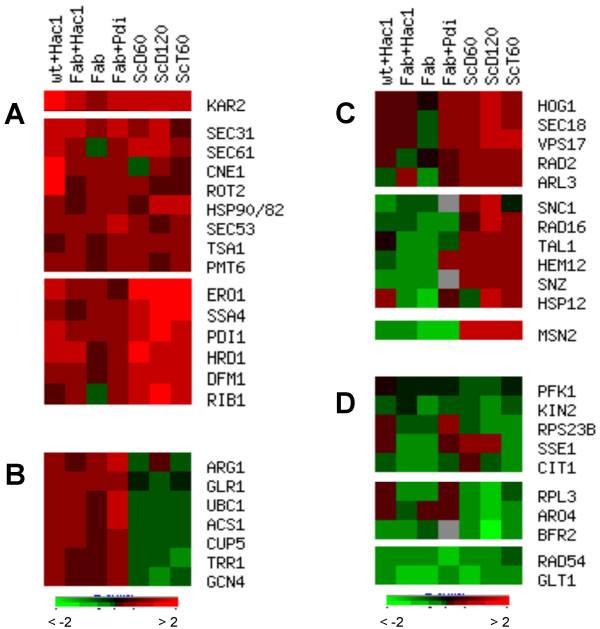
**Comparison of the UPR response in *P. pastoris *and *S. cerevisiae***. Abbreviations for *P. pastoris *strains are explained in Table 3, all data are derived from comparison to the wild type. Data from *S. cerevisiae *were taken from [21], where UPR was induced with DTT or tunicamycin. ScD60 (treatment with DTT after 60 min); ScD120 (treatment with DTT after 120 min); ScT60 (treatment with tunicamycin after 60 min), all compared to a non-treated culture. Cluster analysis was made using EPClust [47], Euklidian distance with complete linkage. Subclusters are shown for the following: A: genes induced in both yeasts; B: upregulated in *P. pastoris*, down-regulated in *S. cerevisiae*; C: down-regulated to unchanged in *P. pastoris*, upregulated in *S. cerevisiae*; D: reduced in both yeasts. Subclusters of genes that are unchanged in both organisms are not displayed. The brightest colouring corresponds to the log_2 _regulation ≥ ± 2.

More than a 2-fold induction of genes like *KAR2 *(BiP, 5-fold), *PDI1 *(protein disulfide isomerase, 3-fold), *ERO1 *(Pdi oxidase, 2-fold) and *SEC61 *(part of translocon complex into the ER, 2-fold) was observed in *P. pastoris *(Figure 1A), analogous to the UPR response described for *S. cerevisiae*[21]. Strong enhancement in the transcription of genes involved in the ER quality control and glycosylation such as *CNE1 *(calnexin), *ROT2 *(glucosidase II) and *SEC53 *(phosphomannomutase) was predominant in *P. pastoris*, while components of the ER-associated protein degradation (ERAD) represented by *HRD1 *and *UBC1 *showed a more consistent upregulation, as compared to *S. cerevisiae*. Also the down-regulation of core metabolism genes such as *PFK1 *(glycolysis), *CIT1 *(TCA cycle), *GLT1 *and *ARO4 *(amino acid biosynthesis) and ribosomal genes (*RPS23B*, *RPL3*) during UPR induction seems to be a common feature for both of the yeasts (Figure 1D), while the transcription of other genes is differentially regulated between the two organisms.

Remarkably, the oppositional regulation of the two general stress markers *GCN4 *and *MSN2 *was noticed. While *GCN4 *was upregulated in *P. pastoris *but down-regulated in *S. cerevisiae*, *MSN2 *behaved the other way round (Figure 1B, C). Induction of *GCN4 *transcription in response to DTT treatment or heterologous protein expression was reported for *T. reesei*, as well as enhanced expression levels of some of its putative target genes related to amino acid biosynthesis, among them *GLT1*, *ARG1*, *ARO4 *[22]. On the contrary to *T. reesei *we can not see a similar behaviour of the amino acid biosynthesis genes included in our marker set, as levels of *GLT1 *(glutamate biosynthesis) are strongly reduced, while *ARG1 *(arginine) transcription is enhanced. Recently, Patil and coworkers [23] described activation of Gcn4p by ER stress and suggested that it acts as an essential transcription factor for the UPR together with or downstream of Hac1p. A link between ER stress and oxidative stress was established in mammalian cells, where the expression of the *GCN4 *homolog ATF4 regulates glutathione biosynthesis and thereby protects the cells against the accumulation of reactive oxygen species deriving from the oxidative protein folding machinery in the ER and the ERAD [24]. Both glutathione (*GLR1*) and thioredoxin (*TRR1*, *TSA1*) related genes show higher expression levels in the Hac1p-overproducing strain compared to the control strain in *P. pastoris *(all displayed in Figure 1A, B).

No direct connection between the general environmental response transcriptional activator Msn2 [25] and UPR regulation has been reported so far. Most significantly, a fraction of genes that are correlated to general stress response appears to be induced in *S. cerevisiae *but remains unchanged to down-regulated in *P. pastoris *along with Msn2 (Figure 1C). Among them Hem12 serves as a marker for oxygen availability, Rad16 and Rad2 belong to the DNA repair mechanism, Arl3 and Vps17 are connected to the transport between the endosomal compartment and the Golgi, while Snooze genes are members of stationary phase related gene family and reported to be induced both by pyridoxal and thiamin depletion [26]. The involvement of the HOG1 (high osmolarity glycerol) pathway in more general environmental stress responses was discussed in [27], and can be connected to the regulation of many other genes including *HSP12 *(all displayed in Figure 1C).

### Induction of the unfolded protein response by heterologous protein secretion

**Figure 2:**
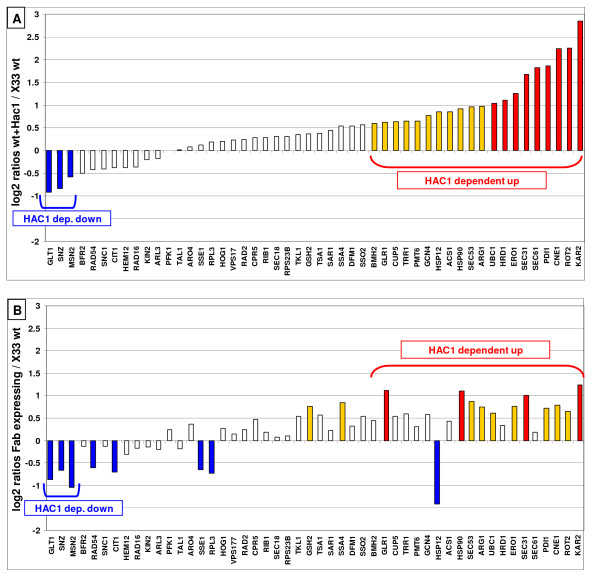
**UPR regulation in *P. pastoris***. A: Log_2 _ratio of Hac1p-overproducing strains compared to the wild type, Hac1p-dependend up- or downregulatated genes are highlighted; B: Log_2 _ratio of cultures producing 2F5 Fab under control of the GAP promoter compared to the wild type, in the same order as A. Red bars: change in transcript up > 2-fold; yellow bars: up > 1.5 fold; white bars: unchanged; blue bars: down > 1.5 fold.

In order to elucidate the behaviour of genes typically known to be under UPR-regulation additionally to other stress-associated markers in recombinant protein producing cells, *P. pastoris *strains secreting the 2F5 antibody fragment under control of the GAP promoter were examined and their response was compared to the Hac1p-overproducing strains in figure 2.

TRAC analysis of these cultures revealed that production of 2F5 Fab actually induces genes identified as UPR-targets before. Genes such as *KAR2*, *PDI1*, *ROT2*, *ERO1*, Calnexin, *SEC31*, and *SEC53 *are significantly upregulated, although not to the same magnitude as in the Hac1p-overproducing strains. Additionally, expression levels of genes belonging to the core metabolism (*GLT1*, *CIT1*), general stress response (*MSN2*, *SNZ*) and ribosomal components (*RPL3*) showed down regulation in the Fab-expressing strain, as has been observed in the strain constitutively overexpressing the UPR transcription factor Hac1p. Out of 20 genes detected to be under Hac1p-specific activation, 11 appeared to be up-regulated in the Fab-expressing strain, and all of the three genes identified to be under Hac1p-dependent down-regulation showed lower transcription levels also in the recombinant strain. No explanation can be given for the divergent regulation pattern of *HSP12*.

Previous transcriptional studies in *Aspergillus nidulans *and *T. reesei *are in agreement with our finding that changes in mRNA levels are more subtle and specific in recombinant protein producing strains than in cultures treated with secretion blockers like DTT, tunicamycin and Brefeldin A or strains with constitutively activated UPR [28,22].

### No reduction of UPR activation due to *PDI1 *overexpression

**Figure 3:**
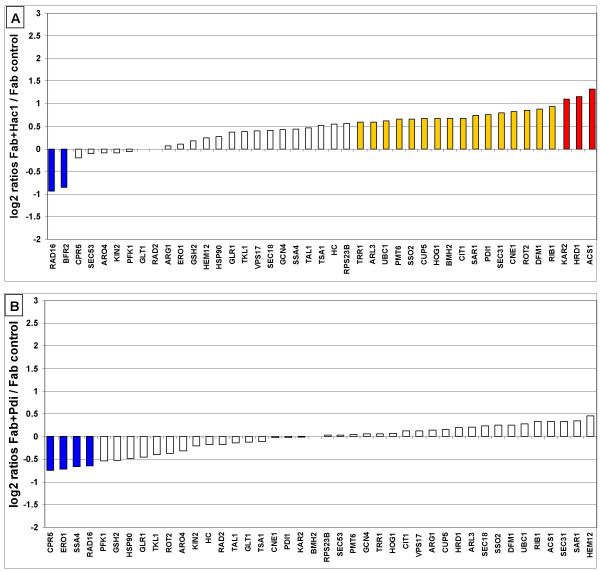
**Effect of engineered folding factors on the transcriptional level**. A: Constitutively UPR induced 2F5 Fab producing SMD1168 (Fab+Hac1) compared to its control strain (containing only the Fab expression cassette). B: 2F5 Fab producing SMD1168 co-expressing *S. cerevisiae PDI1 *compared to the control strain. Both diagrams are ordered from the lowest to the highest log_2 _ratio. Colour legend as in figure 2.

Furthermore, folding engineered strains constitutively co-producing *S. cerevisiae *Pdi1 or Hac1p, which were identified to increase 2F5 Fab secretion 1.9- and 1.3- fold, respectively [7], were subjected to TRAC analysis and compared to their parental strain. The results are displayed in figure 3.

Out of 20 transcripts established to be up-regulated in response to UPR activation, 12 can be found to be at least 1.5-fold induced in the Hac1p-expressing strain as compared to the strain expressing only 2F5 Fab (Figure 3A). The genes detected to be down-regulated in the Hac1p-overexpressing strain as compared to the wild-type show no regulation here as they also have significantly lower expression levels in the Fab expressing strain (compared to the wild type).

Interestingly, when analysing the *PDI1*-overexpressing strains (Figure 3B), we found that most genes seem to be unaffected by *PDI1 *overexpression at a transcriptional level, although 2F5 Fab secretion rates are enhanced under these conditions. Unlike Xu et al. [29] who reported a relief of UPR induction caused by recombinant protein expression when *PDI1 *is overexpressed in *S. cerevisiae*, our results suggest that overexpression of *S. cerevisiae PDI1 *does not alleviate UPR stress in *P. pastoris*. Thus it is likely that there are other mechanisms easing the secretion of the antibody fragments. Interestingly, there are several genes belonging to the vesicular protein transport system involved in ER to Golgi and Golgi to plasma membrane transport (*SAR1*, *SEC18*, *SEC31*, *SSO2*), and the ERAD among the genes most induced (although less than 1.5-fold) in the Pdi1p-coproducing strain, however, some genes connected to Pdi1 like *ERO1 *and the cyclophilin type peptidyl-prolyl isomerase *CPR5 *have significantly lower transcription levels in this strain.

### Influence of cultivation temperature on protein secretion rates and transcriptional response

After monitoring the transcriptional effects specific to UPR induction on a chosen gene set in *P. pastoris*, we intended to uncover the transcriptional responses occurring in cells under production conditions. The particular interest of this collaborative work was to study the interactions between cellular responses triggered by protein overproduction (with particular emphasis on protein synthesis, folding and secretion and related stress responses), and different cultivation temperatures as an example for environmental (cultivation process) conditions.

As our usual production processes were set up at 25°C [4], we intended to investigate production characteristics at an even lower, but still feasible temperature of 20°C. Two independent chemostat cultivations of a *P. pastoris *strain secreting 2F5 Fab under control of the GAP promoter have been conducted, with a dilution rate of D = 0.043 h^-1 ^and glucose as carbon source, one starting at 25°C and shifted to 20°C after 5 volume changes, the other vice versa. In both experiments the biomass reached approx. 25 g/L YDM at both temperatures, however, Fab secretion levels were elevated on average 1.4-fold at the lower temperature (Table 2). The difference was determined to be highly significant by a Student t-test (p = 0.00966).

**Table 2:** Chemostat cultivations of *P. pastoris *X33 producing 2F5 Fab utilizing the GAP promoter at two different temperatures.

	**YDM [g/L]**	**product [mg/L]**	***q*_P _[mg g^-1 ^h^-1^]**
**Chemostat 1**			
**25°C**	25.1	5.3	0.0079
**20°C**	23.2	7.4	0.0122
**Chemostat 2**			
**20°C**	27.0	7.0	0.0091
**25°C**	24.8	5.2	0.0074
**mean ratio 20°C/25°C**	1.0	1.4	1.4

Chemostat 1 was cultivated at 25°C first and temperature was lowered to 20°C after 5 volume changes, chemostat 2 was performed vice versa. Steady state conditions were assumed after 5 volume changes. Biomass (YDM), 2F5 Fab concentration (product) and specific production rate *q*_P _are given for both cultivations, additionally to the mean ratio of 20°C to 25°C for each of these parameters.

Similar improvement of secretion of a single chain fragment (scFv) upon a reduction to 20°C (compared to 30 and 37°C) was reported for *S. cerevisiae *[30], while Li et al. [16] described a positive effect of lower temperature (23°C) on growth and viability during the expression of a herring antifreeze protein in *P. pastoris*, but it should be considered that their data result from shake flasks only. In our experiments, viability remained constantly high (> 98.5 %) at both temperatures examined.

**Figure 4:**
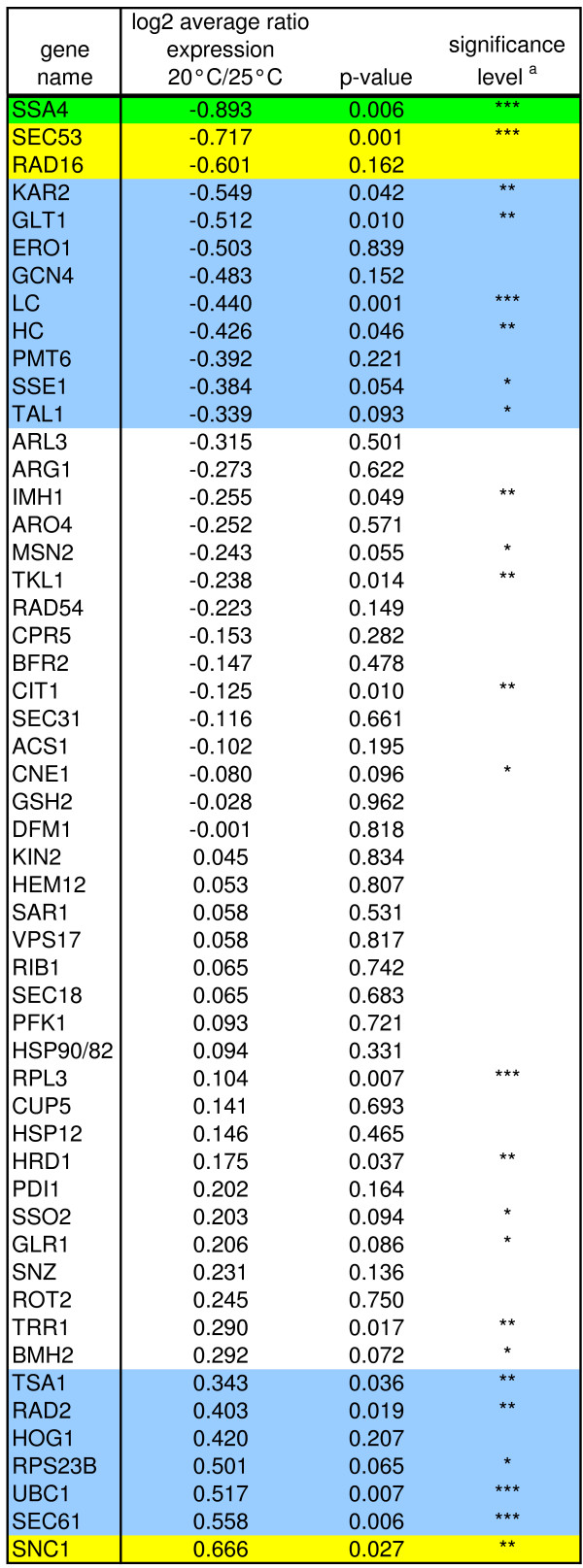
**Comparison of marker genes expression of 2F5 producing *P. pastoris *during steady state conditions**. Log_2 _ratios of the average gene expression between 20 °C and 25 °C in chemostat cultivation (D = 0.043 h^-1^). Genes with ratios exceeding ± one standard deviation (SD) are marked in light blue, ± two SD in yellow and ± three SD in green. The *p*-value (χ^2^-test) is given for each individual marker gene. (a) *** significance level *p *≤ 0.01; ** significance level *p *≤ 0.05; * significance level *p *≤ 0.1

Although the transcriptional levels of the product genes (2F5 Fab light and heavy chain) were reduced at 20°C compared to cultivation on 25°C (Figure 4), specific productivity of the 2F5 Fab protein was significantly increased during the chemostat process at lower temperature (1.4-fold on average). Several genes related to protein targeting to the ER and folding (*SSA4*, *SEC53*, *KAR2*, *ERO1*) and core metabolism genes can be found among the genes down-regulated at 20°C. Transcription of genes involved in the regulation of vesicular transport, exocytosis, ER-associated protein degradation as well as markers for response to oxidative and hyperosmotic stress was enhanced in comparison to 25°C steady state (see Figure 4).

**Figure 5:**
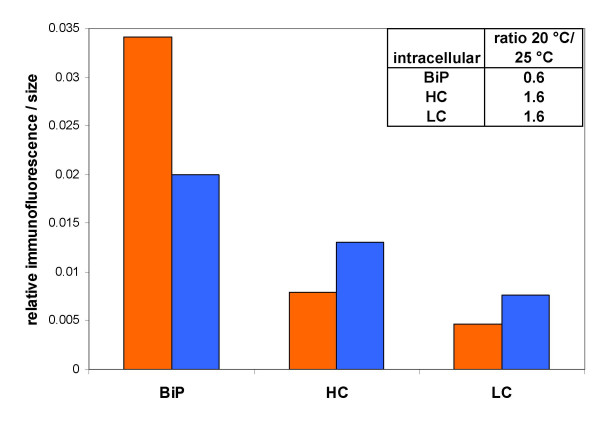
**Intracellular product retention and BiP development analysed by immunofluorescent staining and flow cytometer analysis during cultivation at two different temperatures**. Orange bars: relative fluorescence intensities per cell size obtained at 25 °C steady state; Blue bars: 20 °C steady state. BiP: intracellular signals for the UPR marker BiP/Kar2p (detected with anti-Grp78/BiP specific IgG). HC: intracellular signals for Fab heavy chain (obtained with anti-h-Fab specific IgG); LC: intracellular signals for light chain (analyzed with anti-kappa light chain IgG).

Since there was strong evidence that (partially) unfolded product was retained inside the cells during fed batch cultivations [4,7], thus leading to an upregulation of the UPR marker protein BiP, we investigated the behaviour of these proteins also in the chemostat. According to immunofluorescent staining and flow cytometer analysis intracellular product levels (Fab light chain and heavy chain fragment) are increased at the lower temperature (Figure 5), on the contrary to BiP which shows the same trend as its transcript *KAR2*. Our hypothesis is that at the lower temperature less folding stress is provoked by the native *Pichia *proteins and consequently less cumulative pressure is imposed on the folding machinery. Therefore no induction of BiP transcription could be seen at the lower temperature although more of the recombinant protein was accumulated intracellularly.

ER associated protein degradation is a process detecting misfolded proteins in the ER and redirecting them to the translocon for retranslocation to the cytosol, where they are subjected to proteasomal degradation. Additionally, excess subunits of multimeric proteins that are unable to assemble are degraded through the ERAD mechanism [31]. With *HRD1*, coding for an ubiquitin-protein ligase, that is able to recruit ubiquitin-conjugating enzymes such as the gene product of *UBC1 *next to the translocon pore complex (Sec61p being an essential subunit of the complex), three essential components of the ERAD pathway are up-regulated in correlation to higher protein secretion rates. One could speculate that increased ERAD activity at lower temperature might lead to degradation of excess HC, thus reducing ER stress conditions and therefore leading to better secretion. However, no evidence is given that excess HC is degraded, as intracellular product levels are increased at the lower temperature. No components of the proteasomal system have been included into the study.

All analysed members of the Hsp70-family of chaperones are less transcribed at the lower temperature. This performance can be explained in concordance with their stringent requirement in conditions of heat shock. Together BiP/Kar2p and the Ssa-proteins (ER-localized and cytosolic hsp70, respectively) are required for the import of nascent polypeptides through the translocon into the ER lumen, where BiP is acting as a chaperone. BiP is also necessary for the degradation of misfolded proteins. However, so far no essential role of the Ssa-proteins in the retrograde transport of misfolded soluble proteins during ERAD has been identified [32,33]. The chaperonines of the Sse/Hsp110 subclass of the Hsp70 family, that are encoded by *SSE1 *and *SSE2*, assist in folding by binding to nascent peptides and holding them in a folding-competent state, however, they can not actively promote folding reactions.

Other transcripts significantly down-regulated at the lower temperature belong to the phosphomannomutase *SEC53*, the protein O-mannosyltransferase *PMT6*, both being involved in glycosylation of secretory proteins as well as to the ER resident thiol-oxidase *ERO1 *(acting as a specific oxidant for protein disulfide oxidase). The product of the *IMH1*/*SYS3 *gene is a member of the peripheral membrane Golgins involved in vesicular transport between the late Golgi and a prevacuolar, endosome-like compartment [34].

The transcriptional activator of amino acid biosynthetic genes in response to amino acid starvation *GCN4 *is downregulated 1.4-fold at 20°C steady state levels. Although it might be speculated that a lower concentration of Gcn4 can lead to lower transcription of amino acid biosynthesis genes, in our experiment only *GLT1 *(glutamate synthase) is significantly reduced. The pathways for aromatic amino acids (with *ARO4 *as marker) and arginine biosynthesis (*ARG1*) tend to be downregulated. Also the pentose phosphate shunt (represented by the genes for transaldolase *TAL1 *and transketolase *TKL1*) and the transcription of citrate synthase (*CIT1*) – the rate-limiting enzyme of the TCA cycle – are less active at 20°C. This reduction in transcriptional activity of the core metabolism is a likely explanation for the reduced mRNA levels of the product genes (LC and HC), which are under control of the glycolytic GAP (glyceraldehyde 3-phosphate dehydrogenase) promoter. Essentially the same regulation pattern of amino acid biosynthesis genes and core metabolism genes was observed in a non-expressing control strain cultivated under the same conditions (data not shown). Interestingly, the transcript levels of the ribosomal proteins (*RPL3*, *RPS23*) are raised in both the Fab expressing and the non-expressing strain at 20°C.

Unlike the general transcriptional activator in response to different stresses, *MSN2*, which shows reduced transcription, the genes coding for the key regulatory enzymes of both the cell redox homeostasis (thioredoxin reductase *TRR1*, thioredoxin peroxidase *TSA1*, glutathione oxidoreductase *GLR1*) and osmoregulation (mitogen-activated protein (MAP) kinase *HOG1*) are induced at the lower temperature. This seems to be a more general effect of the reduced temperature as the same regulation pattern can be observed in the *P. pastoris *control strain cultivated under the same conditions (data not shown). Recently, activation of the HOG pathway upon a downshift in temperature (30 to 12°C) was monitored in *S. cerevisiae*, whereas in *Schizosaccharomyces pombe *the Sty1p MAP-kinase (the Hog1p homolog) lead to the activation of several cold-induced genes [35,36]. These authors conclude that the HOG pathway is regulated by changes in membrane fluidity, and that the downward shift in temperature results in the accumulation of glycerol in the yeast cells, while having no major effect on cell viability. Furthermore an increase in intracellular hydrogen peroxide and subsequent induction of anti-oxidant genes was revealed in response to a temperature downshift from 30 to 10°C in *S. cerevisiae *[37]. These results suggest that the cells are subjected to (mild) oxidative stress after cultivation in a low temperature environment, a phenomenon also shared with plants like *Arabidopsis thaliana *[38].

Also the transcript of another regulatory enzyme, the 14-3-3 protein Bmh2, is elevated at the lower temperature, at which higher rates of secretion were observed. 14-3-3 proteins, encoded by *BMH1 *and *BMH2*, were identified to participate in multiple steps of vesicular trafficking, especially in protein exit from the ER, forward trafficking of multimeric cell surface membrane proteins [39] as well as in retrograde transportation within the Golgi apparatus. In *S. cerevisiae *the induction of *BMH1 *has been reported to reduce transcription of folding related genes such as *SSA1/2*, *ERO1*, *HSP 82*, *KAR2 *and *CPR5 *upon a shift from 37°C to 25°C [27], which may also be the case in our experiments.

The yeast syntaxin homologs Sso1 and Sso2 are necessary for the fusion of secretory vesicles to the plasma membrane by acting as target (t-SNARE) for the vesicle membrane receptor protein (v-SNARE) Snc1. Both transcripts were identified to be up-regulated at the reduced temperature under production conditions.

Two genes belonging to the DNA damage response show significantly altered expression upon the temperature reduction; however, the DNA endonuclease (*RAD2*) is induced while *RAD16 *is down-regulated. Detailed functions of proteins involved in the nucleotide excision repair in yeasts are reviewed by [40], however, their connection to cultivation temperature or protein secretion remains elusive.

Generally, the reduction of temperature from 25°C to 20°C does not trigger a transcriptional reaction as definite as the overproduction of the UPR transcription factor Hac1p. As displayed in Figure 4, lowering temperature leads to a regulation pattern that is partially divergent from the UPR specific regulation, with a special discrepancy of single genes such as the HSPs. Interestingly, genes of the core metabolism, amino acid biosynthesis and ribosome biosynthesis, as well as redox and osmotic stress response, were regulated similarly in the Fab-expressing *P. pastoris *strain and a non-expressing control strain. On the other hand, genes belonging to the protein secretory pathway remained unchanged in the control strain, while being significantly regulated in the Fab expressing strain.

Two regulation patterns common to adaptation upon temperature changes were identified also to be conserved in *P. pastoris*. It becomes obvious that the effects caused upon temperature reduction are not ostensibly UPR dependent, but that this regulation is overlapped by alternative (yet unidentified) mechanisms of regulation leading to the final enhancement of antibody fragment secretion.

## Conclusion

Our results indicate the importance of monitoring transcriptional regulations during recombinant protein production processes in real production strains instead of model organisms, even if there are no DNA microarrays available. The transcriptional profiling method TRAC proved to be a valuable tool to analyse genomic responses of a subset of marker genes in *P. pastoris*, and to compare them to those observed in *S. cerevisiae*. Upon UPR induction a lot of markers showed similar transcriptional regulation in both yeasts, however, a significant fraction of transcript levels of genes belonging to general stress response are behaving in an opposite way. Both the effects of *S. cerevisiae *Hac1p and Pdi1 overexpression in *P. pastoris *was studied, additionally to the responses induced by secretion of 2F5 Fab antibody fragment.

Interactions between growth temperature as an example for environmental conditions and specific productivity could be revealed at a transcriptional level. Reduction in temperature led to increased product secretion rates, which are the result of complex regulatory mechanisms and not only influenced by thermodynamic differences in protein folding. Common transcriptional responses to reduced temperature such as induction of genes related to oxidative and osmotic stresses are overlapped by responses to recombinant protein production such as the UPR and the ERAD. Especially the enhanced transcription of components of the secretory pathway became obvious under conditions of higher protein secretion. It is tempting to speculate that at lower temperature a reduced amount of folding stress is imposed on the cells, consequently leading to a higher rate of correctly folded product.

## Methods

### Strains and vectors

GS115 is a histidin auxotrophic *P. pastoris *strain, X33 is its respective histidin prototrophic revertant, and SMD1168 is deleted for the vacuolar protease *pep*4. The construction of *P. pastoris *strain X33 containing both the 2F5 Fab light and heavy chain genes under control of the constitutive GAP (glyceraldehyde-3-phosphate dehydrogenase) promoter, with the *S. cerevisiae *alpha-mating factor signal sequence for secretion, was described in [7]. Similarly, the folding factor engineered *P. pastoris *strains SMD1168 constitutively overexpressing either *S. cerevisiae PDI1 *or the induced form of Hac1p additionally to the Fab or the respective control strains were characterized in [7]. For the evaluation of UPR specific induction in *P. pastoris*, both a SMD1168 and a GS115 were transformed with the UPR transcription factor Hac1p from *S. cerevisiae*, without containing the genes for the Fab. The untransformed *P. pastoris *strain X33 was used for wild type comparison. A summary of all the strains used in this study can be found in Table 3.

**Table 3:** Summary of all the *P. pastoris *strains used in this work.

**Parental strain**	**Product genes**	**Engineered folding factor**	**Abbreviation**
X33			wild type (wt)
GS115		HAC1	wt+Hac1
SMD1168		HAC1	
SMD1168	2F5 Fab		Fab (control)
SMD1168	2F5 Fab	HAC1	Fab+Hac1
SMD1168	2F5 Fab	PDI1	Fab+Pdi
X33	2F5 Fab		for chemostat

GS115 is a histidin auxotrophic *P. pastoris *strain, X33 is a histidin prototrophic revertant, and SMD1168 is deleted for the vacuolar protease *pep*4. All expressing strains carry both the 2F5 Fab light chain gene and the heavy chain fragment under control of the GAP promoter, with the *S. cerevisiae *mating factor alpha leader as secretion signal. The folding factor engineered strains were transformed with the *S. cerevisiae PDI1 *or the induced form of Hac1p under control of the GAP promoter within the *HIS4 *locus.

### Shake flask cultures

5 mL YP-medium (10 g/L yeast extract, 20 g/L peptone) containing 20 g/L glucose were inoculated with a single colony and grown overnight at 30°C. Aliquots of these cultures corresponding to a final OD_600 _of 0.6 were transferred to 10 mL main culture medium (per liter: 10 g yeast extract, 10 g peptone, 100 mM potassium phosphate buffer pH 6.0, 13.4 g yeast nitrogen base with ammonium sulfate, 0.4 mg biotin) and incubated at 30°C at vigorous shaking. After 24 h, 1 mL of these cultures was transferred into fresh 25 mL of main culture medium, and incubated again at 30°C at vigorous shaking for 6.5 h to retain the cells in exponential growth phase, before cells were harvested by centrifugation at 10000 × g for 6 min at 4°C. The pellet was stored at -70°C immediately.

### Chemostat cultivations

The chemostat cultivations were performed in a 2.0 L working volume bioreactor (MBR, Wetzikon, Switzerland) with a computer based process control (ISE, Vienna, Austria) as described in [41].

A shake flask containing 100 mL of YPG medium (per liter: 10 g yeast extract, 10 g peptone, 10 g glycerol) was inoculated with one 1.8 mL cryovial of the *P. pastoris *cell bank, and incubated at 28°C for approximately 24 hours with agitation at 180 rpm. This culture was used to inoculate the starting volume (1.4 L) of the bioreactor to an optical density (OD600) of 1.0.

Cultivation temperature was controlled either at 20°C or 25°C, the pH was controlled at pH 5.0 with 25% ammonium hydroxide and the dissolved oxygen concentration was maintained above 20% saturation by controlling the stirrer speed between 600 and 1200 rpm, whereas the airflow was kept constant at 100 L h^-1^.

The media were as follows:

Batch medium contained per liter:

2.0 g citric acid, 12.4 g (NH_4_)_2_HPO_4_, 0.022 g CaCl_2_· 2H_2_O, 0.9 g KCl, 0.5 g MgSO_4_· 7H_2_O, 40 g glycerol, 4.6 ml PTM1 trace salts stock solution. The pH was set to 5.0 with 25 % HCl.

Chemostat medium contained per liter:

55 g glucose· 1H_2_O, 2.5 g KCl, 1.0 g MgSO_4_· 7H_2_O, 0.035 g CaCl_2_· 2H_2_O, 21.8 g (NH_4_)_2_HPO_4 _and 2.4 ml PTM1 trace salts stock solution; furthermore the pH was set to 5.0 with 25% HCl.

PTM1 trace salts stock solution contained per liter:

6.0 g CuSO_4_· 5H_2_O, 0.08 g NaI, 3.0 g MnSO_4_· H_2_O, 0.2 g Na_2_MoO_4_· 2H_2_O, 0.02 g H_3_BO_3_, 0.5 g CoCl_2_, 20.0 g ZnCl_2, _65.0 g FeSO_4_· 7H_2_O, 0.2 g biotin and 5.0 ml H_2_SO_4 _(95 %-98 %). All chemicals for PTM1 trace salts stock solution were from Riedel-de Haën (Seelze, Germany), except for biotin (Sigma, St. Louis, MO, USA), and H_2_SO_4 _(Merck Eurolab).

After approximately 24 hours the batch was finished and a flow rate of F = 60.0 g h^-1 ^of the dilution pump as well as of the harvest pump was started to adjust a dilution rate of D = 0.0429 h^-1^. The continuous fermentation was performed at least for 5 resident times τ to reach steady state conditions. Then the temperature was shifted to the other set point. Samples were taken after 3 and 5 τ and analyzed as described below.

### Optical density

The samples were diluted in ddH_2_O up to 1:500 to measure the OD at 600 nm.

### Biomass determination

2 × 5 ml culture were collected by centrifugation and the supernatants frozen for further analysis. The pellets were washed in ddH_2_O, centrifuged again, then the pellets were resuspended in ddH_2_O, transferred to a weighed beaker, dried at 105°C until constant weight.

### Quantification of Fab (ELISA)

To determine the Fab content, 96 well microtiter plates (MaxiSorb, Nunc, Denmark) were coated with anti-hIgG (Fab specific) overnight at RT (1:1000 in PBS, pH 7.4), before serially diluted supernatants of *P. pastoris *cultures secreting 2F5 Fab starting with a 1:100 dilution in dilution buffer (PBS adjusted to pH 7.4 containing 0.1% Tween 20 and 1% BSA) were applied and incubated for 2 h at RT. Fab of normal IgG (Rockland Biopharmaceuticals) was used as a standard protein at a starting concentration of 200 ng/mL. After each incubation step the plates were washed four times with PBS containing 1% Tween 20 adjusted to pH 7.4. 100 μL of anti-kappa light chain – AP conjugate as secondary antibody (1:1000 in dilution buffer) were added to each well, and incubated for 1 h at RT. After washing, the plates were stained with pNPP (1 mg/ml p-nitrophenyl phosphate in coating buffer, 0.1 N Na_2_CO_3_/NaHCO_3_; pH 9.6) and read at 405 nm (reference wavelength 620 nm).

### Immunofluorescent staining

Ethanol fixation and immunofluorescent staining were performed as described by [42]. Briefly, fixed cells were washed, blocked in Tris buffer containing 0.1% Triton X-100 and 2 % BSA (both Sigma) and incubated in 200 μl of this buffer and goat anti-hIgG (Fab specific) or goat anti-kappa light chain (both antibodies were applied as FITC conjugates and diluted 1:100). For the detection of intracellular BiP, rabbit anti-Grp78 (StressGen, Vancouver, BC, Canada) was used as described above. Anti-rabbit IgG – FITC conjugate (diluted 1:100) was used as secondary antibody. Finally cells were centrifuged and resuspended in 200 μl PBS, pH 7.4.

### FCM measurements

Cells were analysed on a FACS Calibur (Becton Dickinson, Franklin Lakes, NJ USA) with a 488 nm Argon laser and a 630 nm diode laser. 10^4 ^cells were measured per analysis, using PBS as the sheath fluid. Immunofluorescent staining as well as total protein staining with FITC were measured through a 530/30 BP filter (FL1). Threshold settings were adjusted so that cell debris was excluded from data aquisition.

Immunofluorescence data were normalized to the cell size by dividing the FL1 fluorescence signals by their respective volume-corrected forward scatter signals (FSC) as illustrated in equation (1) [42], and calculating the median of these ratios.

rel.fluorescence=FL1FSC(32)
 MathType@MTEF@5@5@+=feaafiart1ev1aaatCvAUfKttLearuWrP9MDH5MBPbIqV92AaeXatLxBI9gBaebbnrfifHhDYfgasaacH8akY=wiFfYdH8Gipec8Eeeu0xXdbba9frFj0=OqFfea0dXdd9vqai=hGuQ8kuc9pgc9s8qqaq=dirpe0xb9q8qiLsFr0=vr0=vr0dc8meaabaqaciaacaGaaeqabaqabeGadaaakeaacqWGYbGCcqWGLbqzcqWGSbaBcqGGUaGlcqWGMbGzcqWGSbaBcqWG1bqDcqWGVbWBcqWGYbGCcqWGLbqzcqWGZbWCcqWGJbWycqWGLbqzcqWGUbGBcqWGJbWycqWGLbqzcqGH9aqpdaWcaaqaaiabdAeagjabdYeamjabigdaXaqaaiabdAeagjabdofatjabdoeadnaaCaaaleqabaWaaeWaaeaadaWcaaqaaiabiodaZaqaaiabikdaYaaaaiaawIcacaGLPaaaaaaaaaaa@4D54@

### RNA isolation

The frozen cell pellet was resuspended in 1 mL of TRIzol (Invitrogen) and 500 μL of acid washed glass beads (425–600 μM diameters, Sigma) were added. Cells were disrupted with a FastPrep cell homogenizer (Thermo, Germany) using 6 m/s for 2 × 30 s and chilling the cells on ice inbetween. Total RNA was then extracted following the TRI protocol provided by the supplier. Extracted RNA was quantified by measuring OD230/260/280, as well as with the RiboGreen RNA quantitation kit (Invitogen). RNA integrity was checked with the Agilent Bioanalyzer and RNA 6000 Nano Assay kit (Agilent Technologies, California).

PolyA RNA concentration in the extracted total RNA samples was determined with the TRAC protocol described below without the addition of the specific detection probes, and measured with the RiboGreen RNA quantitation kit (Invitrogen).

### Oligonucleotide detection probes

The transcript levels of 55 genes have been chosen as markers, with a great emphasis on folding/secretion related genes as well as general physiological markers. Apart from literature [21], our selection was based on previous microarray experiments [43]. The *P. pastoris *genome sequence data was derived from the ERGO database (Integrated Genomics, Chicago; IL), and the sequences of the gene specific probes are shown in Table 4.

**Table 4:** Marker genes used in TRAC analysis.

**Probe name**	**Gene**	**Location of the probe in CDS**	**Probe length (nt)**	**Tm (°C)**	**GC%**	**Probe Sequence 5'-3'**	**Pool**
ACS1	ERGO:RPPA07570	893–917	25	65.2	48.0	TACTTGGTGGTCAAAAGAGCTCCCA	1
SSE1/2	ERGO:RPPA10049	538–564	27	63.7	44.4	TTTGACGAGCTTCTCAACTGTCCTGTA	1
VPS17	ERGO:RPPA07986	1583–1611	29	64.2	44.8	ACCTGCAGACTGGGTGCTAACTTTTTTCT	1
LC	2F5Fab_light_chain	409–439	31	65.6	41.9	GTACTTTGGCCTCTCTGGGATAGAAGTTATT	1
RPL3	ERGO:RPPA07957	489–521	33	64.9	45.5	TTTTGGTTCAAAGGGGTCTTTCTGATCTGGGTG	1
SEC61	ERGO:RPPA04132	848–882	35	65.4	40.0	CATGATTGGCATGTTGGATGTGTAGAACAATCTGA	1
PDI1	ERGO:RPPA04694	768–804	37	64.3	40.5	GGCTAAAGGGATGTTAGCTTCAGCATATGATTTGAAG	1
TRR1	ERGO:RPPA06201	815–853	39	66.4	46.2	CGAAAACACCTGGAATAGATGTAAGGGAAGAACCTGGGA	1
RIB1	ERGO:RPPA08084	756–796	41	66	39.0	AAGAACGTTTGTCTGCTGGGTGTCTTAGTAAAAGATTTGCT	1
TKL1	ERGO:RPPA06932	1782–1824	43	67.5	39.5	TATTGGAACACCGTCTGGAAGGACTGATAATTGGTAAGATCTA	1
ARG1	ERGO:RPPA07954	813–857	45	70.1	44.4	GTCAAACCTGGAGTTTCATAACAACCTCTGGACTTGATTCCGATG	1
SNZ3/2	ERGO:RPPA10229	557–581	25	63.3	44.0	ATGGCAGCAGCTATCTTTTCAGGAT	2
PMT6	ERGO:RPPA04967	1054–1080	27	62.8	44.4	GTAATCTGTTGTTGGCTGGAACCTTGA	2
GCN4	ERGO:RPPA07905	359–387	29	67.1	48.3	AACGGCTGGAGTGGTAGCAAGAGTAGTTT	2
SSA4	ERGO:RPPA10651	1605–1635	31	65.9	45.2	CATATGATTCCAAGCCATTCTTGGCAGCAAC	2
HC	2F5Fab_heavy_chain	669–701	33	67.9	48.5	GATTTGGGCTCAACTTTCTTGTCCACCTTGGTG	2
KAR2	ERGO:RPPA06939	1325–1359	35	65	40.0	AGTTGGGATAGCAGTGTTTCTGTTGATTAAGGTAG	2
SEC53	ERGO:RPPA08162	349–385	37	63.8	40.5	CGTTTCTGAACTCAATGAATGTTCCTCTTCTGATTGG	2
IMH1	ERGO:RPPA04985	1949–1987	39	62.4	38.5	TGTCTTTTTCCAATTCATCTCGTTTGGAGAGGACTAAAG	2
BFR2	ERGO:RPPA04523	700–740	41	65.8	39.0	AACTCTTGGCTCACAATCTTGCTTTTGTTTAATAGCTTGCC	2
SEC31	ERGO:RPPA06211	1243–1285	43	70	44.2	TTTCGTCGACAAGAGTTTGGTAATCGTTTGTGCTGATGGTGCT	2
HSP90/82	ERGO:RPPA05876	891–915	25	63.9	48.0	CTTAACAGCCAATGGGTCTTCCCAA	3
ARO4	ERGO:RPPA09892	786–812	27	62.8	44.4	TTTCCGTGGGAACAGTCAATCATCAGA	3
ERO1	ERGO:RPPA06115	1531–1559	29	64.4	44.8	TCTCGGTGCCTTTGAGTGCCAATGAAAAT	3
TAL1	ERGO:RPPA08309	550–580	31	63.5	41.9	CCTTGTACCAGTCAAGAATACGTCCAACAAA	3
BMH2	ERGO:RPPA07190	546–578	33	66.1	48.5	AAATGACAGGCGCGGTCAGGAGAGTTTAGAATC	3
ROT2	ERGO:RPPA08267	1634–1668	35	63.3	40.0	ATACGACCTAGTAAGAACAAATGGCCTATGGTTTG	3
GLR1	ERGO:RPPA07699	965–1001	37	67.7	45.9	CTGTTAGATAGTTTTCTACCAGCAGCGATTGCGACAG	3
RAD16	ERGO:RPPA09210	1007–1045	39	63.6	38.5	TATTGTGGGCTTCGTCTAAAATGACCCTATAAAAATGCG	3
GSH2	ERGO:RPPA06484	949–989	41	61.8	39.0	AGAATTTGTTGAACTTTTTTACAACCGCTAAGCTGGGTCAG	3
RAD2	ERGO:RPPA08434	361–403	43	65.8	39.5	CTTGGGTAACCTCATCCGAATCTCTTTGGTCTTTTTTATGTTG	3
CNE1	ERGO:RPPA04260	749–793	45	69.9	42.2	GGATGTACAATTCAGCATCTTCGTTCCAGTTTTCTGGTTTCACAG	3
HOG1	ERGO:RPPA06104	581–605	25	67.8	56.0	TCATCTCGGCGAAAATGCACCCTGC	4
SEC18	ERGO:RPPA05138	1027–1053	27	63.5	40.7	TACCAATATCCAGCTTGTTGGTACCAA	4
CUP5	ERGO:RPPA08088	123–151	29	63.3	41.4	CACTGTATTCTTGATCAGTAAGTCTGGAC	4
SAR1	ERGO:RPPA08441	475–505	31	62.9	38.7	CCTGTCTTAAGTAGATAGAGCAGGTAAATAC	4
ARL3	ERGO:RPPA09566	336–368	33	62.5	42.4	AACGTCCTGTTTATTGGCAAGCATAAGAATCGG	4
DFM1	ERGO:RPPA10235	824–860	37	63.1	40.5	CATTTCTTATCGCAGTAGCTTGGTTTTTCCGTCTTTG	4
HEM12	ERGO:RPPA08255	816–852	37	63.1	40.5	TGGACGGAATAACCAATCCAACGAGACAACATCATAA	4
RAD54	ERGO:RPPA04321	2193–2231	39	63.9	38.5	ACACATGAACTCAATTGCAGTTTAGCTGATTGTCTTTGG	4
RPS23B	ERGO:RPPA09269	187–227	41	66.2	41.5	AACTGAACTCTGACACACTTTCTAATAGCGGAGTTAGGTTG	4
MSN2	ERGO:RPPA05326	985–1027	43	70.5	41.9	GCTCATTCGTGTCTATGGACTTTGCCCTCTTTCTTTGTTTTGT	4
SSO2	ERGO:RPPA06663	356–400	45	71.4	40.0	GCTGTTCTCTGTAGTTGCTTTCAATAATACGGTAGTCCTGAATAG	4
GLT1	ERGO:RPPA07339	1740–1764	25	63.4	48.0	CACAATCGATTCCCTGATGGGATCA	5
UBC1	ERGO:RPPA09581	273–299	27	64.4	44.4	AGAATAGGCGTCCAGGCATTCTTCAAT	5
HSP12	ERGO:RPPA05227	211–239	29	64.8	44.8	TGAACTTGCTCAACTAAGGTAGGTTGGGT	5
PFK1	ERGO:RPPA04164	2447–2479	33	63.4	39.4	TTTTACCCGATTTGTTCTTACCATCGTCTTCCT	5
CIT1	ERGO:RPPA10025	758–792	35	67.1	40.0	GATAGACAGGTATAATCTCATCAGCTCAACGAACT	5
HRD1	ERGO:RPPA05158	1188–1222	35	63.8	42.9	TGAAGGAATTATCGGAATTGGACTGCAGTGGGAAT	5
SNC1	ERGO:RPPA04520	275–313	39	63.9	41.0	AGGAACGATGATAACAATCAGCAAAATCACGATTCCCAG	5
KIN2	ERGO:RPPA04639	640–680	41	70.6	43.9	TTTACGGTTGCATCGTAGTCATCAAATTCTGTCCCGTCAAG	5
TSA1	ERGO:RPPA06990	540–582	43	66.5	44.2	GGACTTGGAAAAGAATTCCTTGGAAGCATCAACTTCTGGCTTG	5
CPR5	ERGO:RPPA09912	314–358	45	71.9	42.2	TGAAGTTCTCGTCCTTGAATCTGCTACCGTAGATAGATTTTCCTC	5

The *P. pastoris *specific probes designed for TRAC, divided into five pools according to their migration in capillary electrophoresis. Location of the probe in the coding sequence (CDS) is indicated. All oligonucleotides are labelled with 6-FAM at 3' and 5' ends.

*P. pastoris *specific oligonucleotide probes were designed by using mathematical algorithms presented in [44]. Criteria used in probe selection were the following: melting temperature, T_m_, limits 60 – 70°C, GC% limits 38 – 62, maximum free energy change in hybridisation ΔG_H _> -15 kcal/mol [45] and minimum target energy change, A_c_, < -10 kcal/mol [46]. A maximum repeat size of 15 nt and maximum similarity of 80% were used as probe specificity criteria. T_m_s were calculated with the nearest neighbour method according to le Novére [45] using 10 nM nucleic acid and 750 mM salt concentrations. The double 6-carboxy fluorescein (6-FAM) labelled oligonucleotides were synthesized by Metabion (Germany).

As the hybridised probes were distinguished from each other based on their different size (ranging from 25 to 45 nucleotides), they had to be assigned into 5 pools such that the probes in the same pool have unique sizes different enough from each other. Quantification of transcript level was according to fluorescence intensity of the respective probes after standardisation.

### TRAC

For the transcriptional profiling, the rapid assay for multiplex transcript analysis based on solution hybridisation of oligonucleotide probes to target mRNA called TRAC presented in [14] was established for *P. pastoris*. Basically, target mRNAs are hybridized with a probe pool consisting of gene-specific oligonucleotide probes with double fluorophore label and biotinylated oligo (dT) and captured on streptavidin-coated magnetic particles. After removal of unbound material, the specific probes are eluted and detected by capillary electrophoresis.

Total RNA corresponding to 100 ng of polyA RNA were hybridized with 4pmol biotinylated oligo (dT) capture probe (Promega) and 1 pmol of each 6-FAM labelled detection probe in hybridisation buffer containing 5 × SSC, 0.2 % (w/v) SDS, 1 × Denhardt solution (0.02% Ficoll, 0.02% polyvinyl pyrrolidone, 0.02% BSA) and 3% dextran sulphate. After 40 min of incubation at 60°C, the 100 μL hybridisation reactions were transferred to a KingFisher 96 magnetic bead particle processor (Thermo Electron) and the following steps of affinity capture, washing and elution were automated in 96-well plates. Alternatively, these steps were performed manually with a 96 well magnetic stand (Promega) and a multichannel pipette (Eppendorf) for one of the temperature shift chemostats.

Affinity capture of the hybridized mRNA targets to 50 μg of streptavidin-coated MyOne Dynabeads (Invitrogen) was followed by several washing steps with decreasing salt concentration to remove unbound material. Briefly, the magnetic beads were washed twice with 1 × SSC, 0.1% SDS for 1.5 min each, then twice with 0.5 × SSC, 0.1 % SDS and finally with 0.1 × SSC, 0.1% SDS once (using 150 μL of the respective washing solution per well). The oligonucleotide probes were eluted to 10 μL of HighFidelity formamide (Applied Biosystems) containing GeneScan-120 LIZ size standard (diluted 1:500, Applied Biosystems) for 20 min at 37°C.

Separation and analysis of the oligonucleotide probes was carried out by capillary electrophoresis with an ABI 3100 Genetic Analyser (Applied Biosystems) and the GeneMapper software.

The individual probes were identified by their migration time (compared to the GeneScan LIZ-120 size standard), and quantified by their peak area. Quantification of transcript level was according to fluorescence intensity of the respective probes after standardisation with signal of the internal standard.

### Internal standard

*E. coli *traT RNA was used as an internal standard in the hybridisations. The following PCR primers (Metabion, Martinsried, Germany) were used to synthesise, from *E. coli *DNA, a template containing the T7 promoter sequence and a 25 nt long T tail: 5' CTAATACGACTCACTATAGGGAGA-ATGAAAAAATTGATGATGGT and 3' TTTTTTTTTTTTTTTTTTTTTTTTT-CAGAGTGCGATTGATTTGGC. The traT RNA was transcribed in vitro by T7-RNA polymerase from this template, using the MEGAscript transcription kit (Ambion, Austin, TX) as recommended by the manufacturer. The synthesized traT RNA was quantified by Agilent Bioanalyser and RiboGreen RNA quantitation kit (Molecular Probes, Leiden, The Netherlands) as recommended by the manufacturer.

To enable the comparison of different samples equal amounts of in vitro transcribed bacterial RNA was added to each sample before the hybridisation, and its respective 6-FAM labelled detection probe was present in each individual probe pool.

### Statistical data evaluation

The relative expression levels given here are based on fluorescence intensity per ng polyA RNA (FL).

TRAC analysis was performed in duplicates for each sample. Variation between these duplicate assays was analysed to calculate a method specific variation (*S*_*MV*_). Additionally, for the temperature experiments, the variation between the two biological replicas, and the different labs (VTT Finland and IAM Vienna) where the TRAC measurements were performed, was determined (*S*_*BV*_). The overall variation (*S*_*A*_) was estimated by the incorporation of error propagation according to the equation (2) of Gauß:

sA=sMV2+sBV2.
 MathType@MTEF@5@5@+=feaafiart1ev1aaatCvAUfKttLearuWrP9MDH5MBPbIqV92AaeXatLxBI9gBaebbnrfifHhDYfgasaacH8akY=wiFfYdH8Gipec8Eeeu0xXdbba9frFj0=OqFfea0dXdd9vqai=hGuQ8kuc9pgc9s8qqaq=dirpe0xb9q8qiLsFr0=vr0=vr0dc8meaabaqaciaacaGaaeqabaqabeGadaaakeaacqWGZbWCdaWgaaWcbaGaemyqaeeabeaakiabg2da9maakaaabaGaem4Cam3aa0baaSqaaiabd2eanjabdAfawbqaaiabikdaYaaakiabgUcaRiabdohaZnaaDaaaleaacqWGcbGqcqWGwbGvaeaacqaIYaGmaaaabeaakiabc6caUaaa@3C02@

For the temperature experiments, the ratio of the FL in 20°C steady state to the FL in 25°C steady state samples was calculated individually for each of the two biological replicas. A Student t-test was performed to assess the probability (*p *values) that the FL 20°C does actually differ significantly from FL 25°C for each marker gene. Finally the mean ratio of the biological replicas was calculated for each probe and a Chi square (χ^2^) -test was performed to combine the individual probabilities obtained from the two independent measurements. The formula is displayed in equation (3), and is based on 2 k degrees of freedom, where k is the number of probabilities being combined. The procedure of significance testing was repeated for each gene.

*χ*^2 ^= -2·∑ln(*p*)

Significant regulation was defined by the ratios being ± one, two or three times the overall standard deviation *S*_*A*_. Simultaneously the significance of FL 20°C being different from FL 25°C was evaluated based on the χ^2 ^distribution resulting in the combined *p *values of the individual t-tests. Each of these two different criteria defines significance based on a different statistical test.

Estimation of the overall variance distributes the variance to all measurements, so that the probability of false negatives increases. To circumvent this problem, the significance of regulations at the single gene level was additionally determined with a t-test. Significant regulation is given if at least one of these criteria is valid. The combined individual probabilities of the two independent chemostat cultivations computed with a χ^2^-test (equation (3)) are stated in Figure 4 along with the mean ratio for each individual marker gene.

## Authors' contributions

BG carried out the TRAC analysis, all data analysis and drafted the manuscript. MM designed and carried out all bioreactor cultivations. JR participated in the computer based design of the TRAC probes and data analysis. MiS participated in selection of the marker genes and design of the study. AB was involved in annotation of the *P. pastoris *genome. MaS participated in selection of the marker genes and design of the study. MP participated in the conception of the study. DM conceived of the study, and participated in statistical analysis and drafting of the manuscript.
